# An Exploration of Traditional Chinese Medicinal Plants with Anti-Inflammatory Activities

**DOI:** 10.1155/2017/1231820

**Published:** 2017-04-04

**Authors:** Chao Fan, Hui-zi Jin, Lehao Wu, Yu Zhang, Richard D. Ye, Weidong Zhang, Yan Zhang

**Affiliations:** School of Pharmacy, Shanghai Jiao Tong University, Shanghai 200240, China

## Abstract

In a continuing effort to discover more anti-inflammatory medicinal plants in China, the anti-inflammatory activities of 101 extracts from different parts of 84 traditional medicinal plants were evaluated by a panel of in vitro and in vivo assays. Nuclear factor-kappa B (NF-*κ*B) inhibitory effects were determined by luciferase assay in stably transfected Hela cells. Cytotoxic activities were assessed using the MTT assay. Inhibitory effects on LPS-induced nitric oxide production and proinflammatory mediators were assessed by Griess reaction and Real-Time PCR analysis, respectively. In vivo anti-inflammatory activities were examined by xylene-induced mice ear edema model. In total, 22 extracts showed promising NF-*κ*B inhibitory effects whereas 9 of them did not affect the cell viability. The 9 hit extracts were active in at least one of the subsequently performed in vitro pharmacological test systems. The extract from* Hemerocallis minor* (root) was selected to perform the in vivo study because it demonstrated significant suppressive effects in all the in vitro assays. Results showed that the extract of* Hemerocallis minor (Root) *was able to alleviate ear edema effectively in xylene-induced mice ear edema mode. Collectively, our study provides evidence for the potential anti-inflammatory effects of the medicinal plants traditionally used in China. Further phytochemical and pharmacological studies remain to be clarified.

## 1. Introduction

Inflammation is an adaptive response of body tissues to external challenge or cellular injury. It is generally thought that inflammation is a beneficial host response defense system, but it can become harmful if dysregulated [1]. The complex mediators including proinflammatory and cytotoxic cytokines, growth factors, bioactive lipids, and nitric oxide in the uncontrolled inflammation reaction can sustain or induce a pathologic process involved in different diseases, such as allergy, sepsis, arthritis, the metabolic syndrome, autoimmune diseases, and cancer [2]. The inhibition of inflammatory mediators on the transcriptional level has emerged as a promising approach for the development of anti-inflammatory drugs [3]. Nuclear transcription factor-kappa B (NF-*κ*B) plays a key role among the involved transcription factors to regulate various genes involved in immune and acute phase inflammatory responses. The activation of NF-*κ*B induces the transcriptions of iNOS, COX-2, TNF-*α*, and proinflammatory cytokines [4, 5]. Therefore, the NF-*κ*B signaling pathway has long been considered the “holy grail” as a target for anti-inflammatory drug [3].

Traditional Chinese medicinal plants work well for many diseases and have been used for centuries in China. Its increasing use in recent years is evidence of a public interest in having alternatives to conventional medicine [6]. A variety of medicinal plants have been reported to have anti-inflammatory properties. However, a comprehensive investigation on exploring more anti-inflammatory medicinal plants and their action mode is lacking. In a continuing effort to discover more medicinal plants with anti-inflammatory effects in China, 101 extracts from different parts of 84 TCMs (Table 1) were screened for their potential inhibitory activities on NF-*κ*B activation. In order to further determine their anti-inflammatory activity, the inhibition of the proinflammatory cytokines (TNF-*α*, IL-6, and IL-1*β*) or the NO production induced in LPS-stimulated murine RAW 264.7 was investigated. Mouse ear edema assay was used to verify the in vivo anti-inflammatory activity of the selected plant extracts.

## 2. Materials and Methods

### 2.1. Plant Material

The plant materials used in this study were collected from different locations of China. The collected species were identified and authenticated by taxonomists from the Second Military Medical University (China). All plant samples were air-dried and finally ground to a fine powder before further processing.

### 2.2. Extraction

The medicinal plants used in this study were collected at the different areas in China. All voucher specimens have been deposited at the Lab. of Chemistry and Bioactivity of TCM, Shanghai Jiaotong University. The air dried powder of medicinal plants was extracted with 95% ethanol three times (24 h × 3) at room temperature. The extract was evaporated in vacuo to leave a residue extract, which was measured and dissolved in DMSO as the final concentration of 10 mg/mL, respectively.

### 2.3. Animals

Male C57BL/6 mice, 6–8 weeks old and weighing approximately 20 g, were purchased from SLACCAS Laboratory Animal Co., Ltd (Shanghai, China). All mice were housed (four to five animals per cage) with a 12/12 h light/dark cycle, with ad libitum access to food and water. The housing, breeding, and animal experiments were in accordance with the National Institutes of Health Guide for the Care and Use of Laboratory Animals, with procedures approved by the Biological Research Ethics Committee of Shanghai Jiao Tong University.

### 2.4. Cell Culture

The RAW 264.7 macrophage cell line was obtained from the China Cell Line Bank (Beijing, China). The cells were cultured in DMEM medium supplemented with 10% FBS, penicillin (100 U/mL), and streptomycin (100 *μ*g/mL) in a 37°C and 5% CO_2_ incubator. The human cervical carcinoma cell line Hela was transfected with pNF-*κ*B-Luc reporter plasmid, which contains five copies of NF-*κ*B binding sequence (Stratagene, La Jolla, CA). The transfected cells were maintained in Dulbecco's modified Eagle's medium (DMEM) supplemented with 10% fetal bovine serum.

### 2.5. NF-*κ*B Transactivation Assay

The examination of NF-*κ*B activity was performed by a modification of methods as described previously [7]. HeLa cells expressing NF-*κ*B-Luciferase were seeded in 96-well plates at a density of 1.5 × 10^4^ cells per well. After cells became adherent, they were serum-starved in DMEM without phenol red overnight before screening assay. On the next morning, cells were pretreated with the investigated samples, positive control (parthenolide, 10 *μ*M), or solvent vehicle (0.1% DMSO in culture medium) for 1 h and stimulated with 5 ng/mL TNF-*α* for 4 h [7]. Afterwards, the cells were lysed with luciferase lysis buffer and the luminescence of the firefly luciferase was measured with the microplate reader (FlexStation 3; Molecular Devices, Silicon Valley, CA, USA).

### 2.6. The MTT Assay for Cell Viability

Cells were plated overnight in 96-well plate (5 × 10^3^/well). Then, the cells were treated with 0.1% DMSO (vehicle control) or the plant extract samples with various concentrations for 24 h. Following another 4 h incubation with 20 *μ*L of MTT solution (5 mg/mL), the culture supernatant was discarded and 100 *μ*L of DMSO was added to each well to dissolve the formazan crystal. OD absorbance was recorded at 490 nm with the microplate reader (FlexStation 3, Molecular Devices, Silicon Valley, CA, USA).

### 2.7. Determination of NO Production

RAW 264.7 macrophages were plated in 48-well plate (1.5 × 10^5^/well). After cells became adherent, they were pretreated with the test samples, positive control (parthenolide, 10 *μ*M), or solvent vehicle (0.1% DMSO in culture medium) for 1 h and then stimulated with 100 ng/mL LPS for additional 12 h. The nitrite accumulated in culture medium was measured as an indicator of NO production based on Griess reaction. Briefly, 50 *μ*L of cell culture medium was mixed with 50 *μ*L of Griess reagent (1% sulfanilamide in 5% phosphoric acid, 1%  *α*-naphthylamide in H_2_O) in a 96-well plate, incubated at room temperature for 10 min, and then measured at 540 nm using a microplate reader (FlexStation 3, Molecular Devices, Silicon Valley, CA, USA) [8].

### 2.8. RNA Extraction and Reverse Transcription-Polymerase Chain Reaction (RT-PCR)

RAW 264.7 macrophages were plated in 12-well plate (5 × 10^5^/well). Cells pretreated with the test samples, positive control (parthenolide, 10 *μ*M), or solvent vehicle (0.1% DMSO in culture medium) for 1 h were incubated with 100 ng/mL LPS for additional 4 h. Total RNA was extracted from cells using Trizol reagent (Invitrogen, Carlsbad, CA, USA). Reverse transcription of RNA was performed with the Reverse Transcription System A3500 kit (Promega, Madison, WI) according to the manufacturer's protocol. Relative quantification of gene expression was performed with SYBR® Green Real-Time PCR Master Mix (TOYOBO, Osaka, Japan) and conducted with the Eppendorf Mastercycler ep realplex (Hauppauge, NY). The following primers were used: TNF-*α* (5′-TTCTCATTCCTGCTTGTGG-3′; 5′-ACTTGGTGGTTTGCTACG-3′), IL-6 (5′-CTTCTTGGGACTGAT G-3′; 5′-CTGGCTTTGTCTTTCT-3′), and IL-1*β* (5′-GATCCACACTCTCCAGCTGCA-3′; 5′-CAACCAACAAGTGATATTCTCCATG-3′). The primers for the mouse housekeeping gene glyceraldehyde-3-phosphate dehydrogenase (GAPDH) were 5′-CCTTCCGTGTTCCTACC-3′ and 5′-CAACCTGGTCCTCAGTGT A-3′ [9].

### 2.9. Xylene-Induced Ear Edema in Mice

Xylene-induced ear edema in mice was initiated according to a previously described method [10]. Briefly, the male mice were randomly divided into four groups (*n* = 6/group). Mice were intraperitoneally (i.p.) given twice vehicle, dexamethasone (3 mg/kg), and two doses (250 and 500 mg/kg) of the test sample 12 and 1 h before xylene treatment. After the final administration, ear edema was induced by applying 20 *μ*L of xylene to the outer and inner surface of the right ear. The left ear served as a control. Mice were sacrificed after 1 h by carbon dioxide suffocation, and both ears were removed. 7 mm diameter ear punch biopsies were collected by using a metal punch and weighed. Edema (Δ*W*) was defined as the difference in weight (mg) between the right and the left ear sample Δ*W* = *W*_R_ − *W*_L_. The extent of the edema was expressed by percentage of increase in the ear tissue weight (%), using the following formula [10]: percentage of edema weight (%) = (*W*_R_ − *W*_L_) × 100/*W*_L_.

### 2.10. Statistical Analysis

The results are expressed as mean ± SEM. Statistical differences were compared with one-way ANOVA. *p* values < 0.05 were considered to be significant. All statistical tests were carried out using the GraphPad Software (California, USA).

## 3. Results

### 3.1. Inhibitory Effects of the Chinese Medicinal Plant Extracts on TNF-*α*-Induced NF-*κ*B Activation

**Figure 1:**
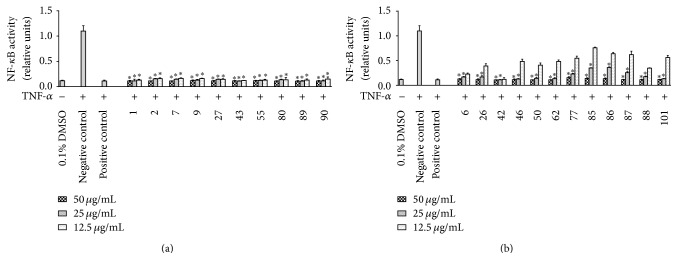
Inhibitory effects of the plant extracts (50 *μ*g/mL, 25 *μ*g/mL, and 12.5 *μ*g/mL) on NF-*κ*B activation in Hela/NF-*κ*B-luc cells. Hela cells were treated with solvent vehicle (DMSO, 0.1%), positive control (parthenolide, 10 *μ*M), or plant extract samples (50 *μ*g/mL, 25 *μ*g/mL, and 12.5 *μ*g/mL) 1 h prior to the addition of TNF-*α* (5 ng/mL), and cells were further incubated for 4 h. The numbers were used to abbreviate plant names (see Table 1 for full names). The produced luciferase activity was assayed as described in* Materials and Methods*. Data points represent mean ± SEM; *n* = 3, ^*∗*^*p* < 0.05 (ANOVA) compared with negative control.

In a primary screening, 101 different extracts from 84 plants were assessed for their potential to inhibit TNF-*α* induced NF-*κ*B activation in Hela cells at the concentrations of 50, 25, and 12.5 *μ*g/mL. As shown in Figure 1(a), 10 out of 101 extracts showed significant NF-*κ*B inhibitory effects at all test concentrations in a dose-dependent manner (*p* < 0.05 compared with negative control). These 10 extracts were including* 1-Rehderodendron macrocarpum (Stem), 2-Styrax suberifolia (Stem), 7-Alangium kurzii (Root), 9-Alangium kurzii *var.* handelii (Leaf), 27-Cleome gynandra (Seeds), 43-Aspidistra omeiensis (Root), 55-Disporum calcaratum (Herb), 80-Liriope spicata (Herb), 89-Paris polyphylla (Flower), *and* 90-Paris polyphylla (Root)*. Twelve out of 101 extracts showed significant NF-*κ*B inhibitory effects (*p* < 0.05 compared with negative control) at the concentrations of 50 and 25 *μ*g/mL (Figure 1(b)). These extracts were including* 6-Alangium chinense *ssp.* triangulare (Stem), 26-Capparis masaikai (Fruit), 42-Aspidistra omeiensis (Leaf), 46-Cardiocrinum cathayanum (Herb), 50-Disporopsis aspera (Herb), 62-Hemerocallis minor (Root), 77-Liriope minor (Herb), 85-Ophiopogon japonicus (Root), 86-Ophiopogon mairei (Herb), 87-Ophiopogon tonkinensis (Herb), 88-Paris chinensis (Herb), *and* 101-Smilacina henryi (Herb).*

### 3.2. Effects of the Extracts on Cell Viability

In order to exclude the potential influence of cytotoxicity, the effects of these 22 effective plant extracts at the concentrations of 50, 25, and 12.5 *μ*g/mL along with parthenolide (2.5*∽*15 *μ*M) on proliferation of Hela cells and RAW 264.7 cells were evaluated using MTT assay. As shown in Table 2, 9 out of 22 extracts did not affect cell viability (>75%) at their effective doses. Parthenolide (10 *μ*M) showed slight effects on cell viability of Hela and RAW 264.7 macrophages (S1 Figure in Supporting Information available online at https://doi.org/10.1155/2017/1231820). These extracts were including* 1-Rehderodendron macrocarpum (Stem), 2-Styrax suberifolia (Stem), 7-Alangium kurzii (Root), 26-Capparis masaikai (Fruit), 46-Cardiocrinum cathayanum (Herb), 62-Hemerocallis minor (Root), 77-Liriope minor (Herb), 80-Liriope spicata (Herb)*, and* 87-Ophiopogon tonkinensis (Herb). *Therefore, the remaining nine plant extracts were selected for further investigation.

### 3.3. Inhibitory Effects of the Extracts on LPS-Induced NO Production in RAW 264.7 Cells

**Figure 2:**
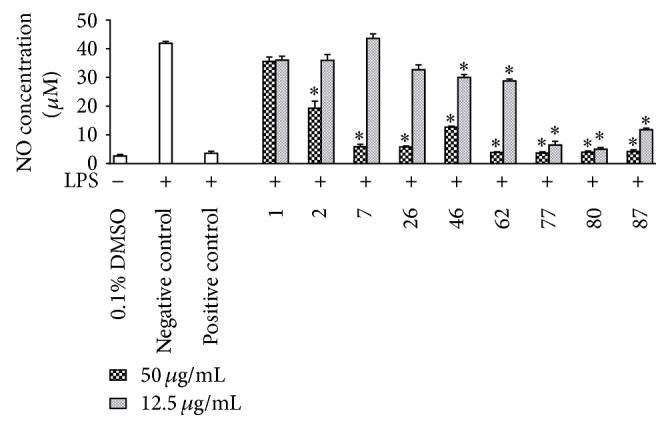
Inhibitory effect of plant extracts (50 *μ*g/mL, 12.5 *μ*g/mL) on NO production in LPS-stimulated RAW 264.7 macrophages. RAW 264.7 cells were treated with solvent vehicle (DMSO, 0.1%), positive control (parthenolide, 10 *μ*M), or plant extract samples (50 *μ*g/mL, 12.5 *μ*g/mL) 1 h prior to the addition of LPS (100 ng/mL), and cells were further incubated for 24 h. The numbers were used to abbreviate plant names (see Table 1 for full names). NO levels were determined with Griess reagent. Data points represent mean ± SEM; *n* = 3, ^*∗*^*p* < 0.05 (ANOVA) compared with negative control.

The inhibitory effects of the nine plant extracts on NO production in LPS-stimulated RAW 264.7 cells were assessed. As shown in Figure 2, five extracts, including* 46-Cardiocrinum cathayanum (Herb), 62-Hemerocallis minor (Root), 77-Liriope minor (Herb), 80-Liriope spicata (Herb), *and* 87-Ophiopogon tonkinensis (Herb),* showed significant inhibitory effects at both low (12.5 *μ*g/mL) and high concentrations (50 *μ*g/mL).

### 3.4. Inhibitory Effects of the Plant Extracts on mRNA Expressions of TNF-*α*, IL-6, and IL-1*β* in LPS-Stimulated RAW 264.7 Cells

**Figure 3:**
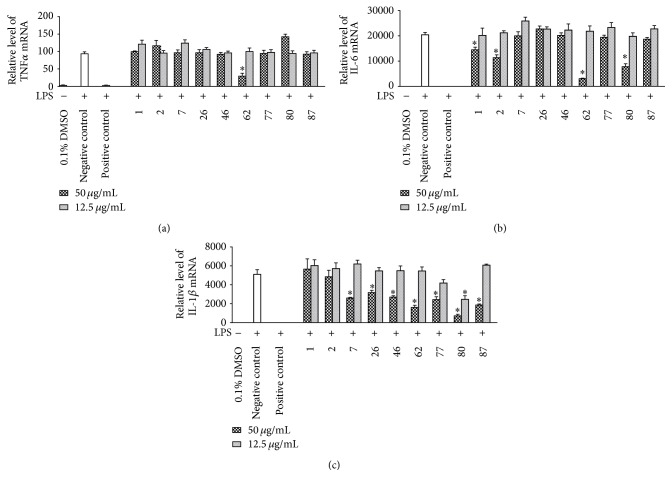
Inhibitory effects of plant extracts (50 *μ*g/mL, 12.5 *μ*g/mL) on LPS-induced mRNA expression of proinflammatory cytokines in RAW 264.7 macrophages. RAW 264.7 cells were treated with solvent vehicle (DMSO, 0.1%), positive control (parthenolide, 10 *μ*M), or plant extract samples (50 *μ*g/mL, 12.5 *μ*g/mL) 1 h prior to the addition of LPS (100 ng/mL), and cells were further incubated for 4 h. The numbers were used to abbreviate plant names (see Table 1 for full names). The mRNA levels of TNF-*α* (a), IL-6 (b), and IL-1*β* (c) were determined by quantitative Real-Time PCR. Data points represent mean ± SEM; *n* = 3, ^*∗*^*p* < 0.05 (ANOVA) compared with negative control.

The effects of the above nine extracts active in both NF-*κ*B and NO assays were also assessed at the mRNA level by RT-qPCR. As shown in Figure 3(a), one extract* (62-Hemerocallis minor)* exhibited the suppressive effect on TNF-*α*. Four extracts including* 1-Rehderodendron macrocarpum (Stem), 2-Styrax suberifolia (Stem), 62-Hemerocallis minor (Root), *and* 80-Liriope spicata (Herb)* and seven extracts including* 7-Alangium kurzii (Root), 26-Capparis masaikai (Fruit), 46-Cardiocrinum cathayanum (Herb), 62-Hemerocallis minor (Root), 77-Liriope minor (Herb), 80-Liriope spicata (Herb), *and* 87-Ophiopogon tonkinensis (Herb) *significantly inhibited the mRNA expression of IL-6* and* IL-1*β*, respectively (as shown in Figures 3(b) and 3(c)). Among these effective extracts, the extract* 62-Hemerocallis minor (Root) *was observed to suppress all the proinflammatory cytokines TNF-*α*, IL-6, and IL-1*β*.

### 3.5. Inhibitory Effects of 62-Hemerocallis Minor (Root) on Xylene-Induced Mice Ear Edema

**Figure 4:**
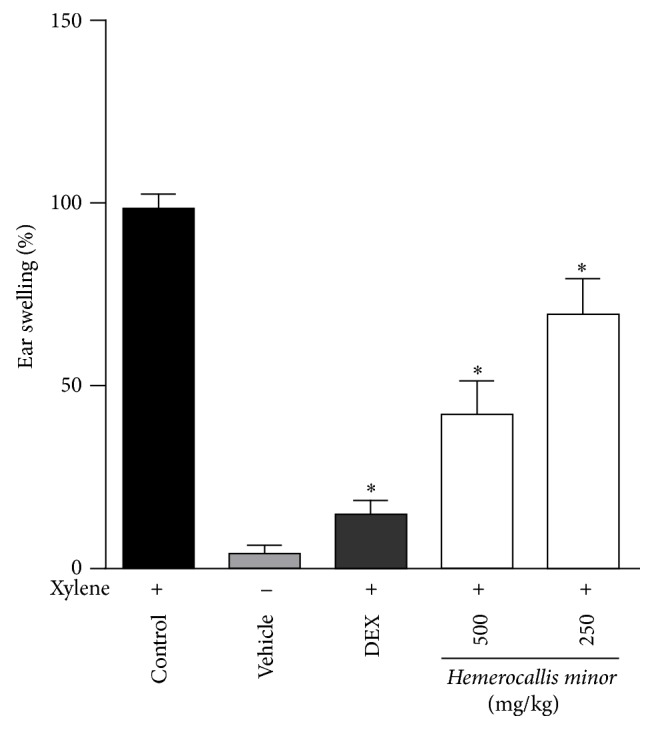
The effect of* Hemerocallis minor* on xylene-induced ear edema in mice. The mice (*n* = 6/group) were given (i.p.)* Hemerocallis minor *plant extracts or 3 mg/kg dexamethasone (DEX), 1 h and 12 h prior to 20 *μ*L xylene treatment. Both ears of each mouse were removed 1 h after 20 *μ*L of xylene applied to the right ear and the edema was calculated as detailed in Section 2.9. The data were expressed as mean ± SEM and the differences between treatment and control were tested by ANOVA. A value of *p* < 0.05(*∗*) was considered statistically significant.

*Hemerocallis minor,* which was effective in all the above in vitro assays, was selected to further evaluate the in vivo anti-inflammatory effect. Xylene-induced mice ear edema model was used in this study. Xylene can stimulate local inflammation and then induce edema of tissues. Edema was determined by the increase in ear weight due to an inflammatory challenge.  Figure 4 exhibited that* Hemerocallis minor* extract was effectively inhibiting ear edema in a dose-dependent manner. Compared with the control group,* Hemerocallis minor* extract at the doses of 250 mg/kg and 500 mg/kg significantly alleviated xylene-induced edema, and the percentage of edema weight was 69% and 42%, respectively. Similarly, dexamethasone (3 mg/kg) led to a percentage of edema weight value of 15%. The results indicated that the* Hemerocallis minor* extract has a significant anti-acute-inflammation activity.

## 4. Discussion

Traditional Chinese medicinal plants play a significant role in drug discovery and development. Many have provided the foundation for modern pharmaceuticals and drug leads [11, 12]. In this study, we collected 101 extracts from 84 species of traditional medicinal plants to assess their potential anti-inflammatory effects in a panel of in vitro and in vivo assays. The purpose of this study is to provide more evidence for the plants which have been recorded with uses linked to anti-inflammatory effects and explore more species of undiscovered medicinal plants with anti-inflammatory properties.

NF-*κ*B controls the expression of genes involved in a number of physiological responses, including immune and inflammatory responses [13]. Given NF-*κ*B's importance in human diseases related to inflammatory disorders and the fact that many anti-inflammatory drugs interfere with NF-*κ*B signaling, we established a screening assay specific for NF-*κ*B activity to discover medicinal plants targeting NF-*κ*B signal pathway [14]. Twenty-two out of 101 extracts exhibit significant inhibitory effects on NF-*κ*B transactivation at concentrations of 25 and 50 *μ*g/mL while ten among them are effective at even lower concentration (12.5 *μ*g/mL). Among these 22 extracts,* Paris polyphylla, Liriope spicata,* and* Cleome gynandra* have been historically used in China and have been exploited from the chemical components to biological activities including anti-inflammation and anticancer [15–17]. Limited information regarding other species of these medicinal plants has been reported. Our study provides the first evidence for their potential anti-inflammatory effects targeting NF-*κ*B signaling pathway. In the present study, we show that the extracts from different species even from the same genus lead to different pharmacological responses. Different clinical application of extracts from the different species of the same genus or different parts of the same plant may be explained by differences in pharmacological responses induced by them.

Macrophage activation by LPS leads to the phosphorylation, ubiquitination, and subsequent degradation of the inhibitory *κ*B (I*κ*B). This induced degradation of I*κ*Bs allows NF-*κ*B to freely enter the nucleus and to induce the production of NO and transcriptions of proinflammatory cytokines including IL-1*β*, IL-6, and TNF-*α*, which are important for the inflammatory and immune response [18, 19]. The pharmacological reduction of LPS-inducible inflammatory mediators is regarded as one of the essential conditions to alleviate acute and chronic inflammations caused by activation of macrophages [20]. Therefore, LPS-stimulated macrophage cells provide us with an excellent model for investigating inflammatory response and subsequently for screening anti-inflammatory drugs and their underlying mechanisms [21, 22]. In the present study, the hit extracts found in the preliminary screening with NF-*κ*B assay and without cellular toxicities were further subjected to detect the effects on the level of NO production and TNF-*α*, IL-1*β*, and IL-6 mRNA expressions in LPS-stimulated RAW 264.7 macrophages. The nine hit extracts were active in at least one of the subsequently performed in vitro pharmacological test systems, which were including NO production and cytokines mRNA expressions.* Hemerocallis minor* extract was found to be effective in all in vitro assays and not to affect the cell viability. The extract from* Hemerocallis minor* also inhibited xylene-induced mice ear edema.* Hemerocallis minor*, frequently referred to as yellow lily, has been applied in China as food and folk remedy, particularly for heat-clearing and breast mastitis [21, 22]. To our knowledge, it is the first report that* Hemerocallis minor *may possess possible therapeutic anti-inflammatory properties. Our results might provide a rationale for the traditional use of this plant in China and make it a highly interesting medicinal plant, having the potential to be further explored as anti-inflammatory agents. However further phytochemical and pharmacological studies on* Hemerocallis minor* remain to be performed.

## 5. Conclusions

In summary, the present study demonstrates that extracts from nine medicinal plants exhibit properties of anti-inflammatory activity by either suppressing NO production or the major proinflammatory cytokines TNF-*α*, IL-1*β*, and IL-6.* Hemerocallis minor* is the particularly interesting one which is effective in all the in vitro and in vivo assays and has the potential to be developed as anti-inflammatory therapeutics or preventatives.

## Supplementary Material

S1 Figure: Cytotoxicity of parthenolide in RAW 264.7 macrophages and Hela cells. Cells were incubated with parthenolid (2.5, 5, 10 and 15 μM) for 24 h. The cell viability was tested by MTT assay. The data were expressed as mean ± S.E.M.

## Figures and Tables

**Table 1:** Overview of the collected plants used in this investigation.

Number	Latin name	Plant part	Sample location
(1)	*Rehderodendron macrocarpum*	*Stem*	Lushan, Jiangxi
(2)	*Styrax suberifolia*	*Stem*	Zhangjiajie, Hunan
(3)	*Alangium chinense*	*Root*	Shijiazhuang, Hebei
(4)	*Alangium chinense*	*Stem*	Chengdu, Sichuan
(5)	*Alangium chinense *ssp.* triangulare*	*Root*	Jingzhou, Hubei
(6)	*Alangium chinense *ssp.* triangulare*	*Stem*	Jingzhou, Hubei
(7)	*Alangium kurzii*	*Root*	Lushan, Jiangxi
(8)	*Alangium kurzii*	*Stem*	Lushan, Jiangxi
(9)	*Alangium kurzii *var.* handelii*	*Leaf*	Lushan, Jiangxi
(10)	*Alangium kurzii *var.* handelii*	*Root*	Lushan, Jiangxi
(11)	*Alangium platanifolium*	*Leaf*	Lushan, Jiangxi
(12)	*Alangium platanifolium*	*Root*	Lushan, Jiangxi
(13)	*Alangium kurzii *var.* umbellatum*	*Stem*	Lushan, Jiangxi
(14)	*Musa acuminata*	*Leaf*	Xishuangbanna, Yunnan
(15)	*Musa balbisiana*	*Flower*	Jingzhou, Hubei
(16)	*Musa balbisiana*	*Root*	Yunwushan, Guangdong
(17)	*Musa basjoo*	*Stem*	Jingzhou, Hubei
(18)	*Musa basjoo*	*Flower*	Shanghai
(19)	*Musa basjoo*	*Leaf*	Xishuangbanna, Yunnan
(20)	*Musa nana*	*Leaf*	Xishuangbanna, Yunnan
(21)	*Musa paradisiaca*	*Flower*	Yunwushan, Guangdong
(22)	*Musella lasiocarpa*	*Herb*	Shanghai
(23)	*Ravenala madagascariensis*	*Leaf*	Xishuangbanna, Yunnan
(24)	*Capparis acutifolia*	*Herb*	Bozhou, Anhui
(25)	*Capparis bodinieri*	*Herb*	Chengdu, Sichuan
(26)	*Capparis masaikai*	*Fruit*	Shijiazhuang, Hebei
(27)	*Cleome gynandra*	*Seeds*	Shijiazhuang, Hebei
(28)	*Ceratostigma minus*	*Herb*	Chengdu, Sichuan
(29)	*Limonium bicolor*	*Herb*	Chengdu, Sichuan
(30)	*Plumbago zeylanica*	*Herb*	Xishuangbanna, Yunnan
(31)	*Stemona sessilifolia*	*Root*	Shanghai
(32)	*Aletris spicata*	*Herb*	Chengdu, Sichuan
(33)	*Allium macrostemon*	*Stem*	Shanghai
(34)	*Allium tuberosum*	*Seeds*	Shanghai
(35)	*Aloe vera*	*Leaf*	Shanghai
(36)	*Anemarrhena asphodeloides*	*Root*	Shanghai
(37)	*Asparagus cochinchinensis*	*Root*	Shanghai
(38)	*Asparagus dauricus*	*Leaf*	Xishuangbanna, Yunnan
(39)	*Asparagus filicinus*	*Herb*	Shanghai
(40)	*Aspidistra caespitosa*	*Leaf*	Lushan, Jiangxi
(41)	*Aspidistra lurida*	*Herb*	Chengdu, Sichuan
(42)	*Aspidistra omeiensis*	*Leaf*	Emeishan, Sichuan
(43)	*Aspidistra omeiensis*	*Root*	Emeishan, Sichuan
(44)	*Aspidistra typica*	*Leaf*	Shanghai
(45)	*Allium victorialis*	*Herb*	Shijiazhuang, Hebei
(46)	*Cardiocrinum cathayanum*	*Herb*	Lushan, Jiangxi
(47)	*Cardiocrinum cathayanum*	*Fruit*	Lushan, Jiangxi
(48)	*Chlorophytum comosum*	*Herb*	Lushan, Jiangxi
(49)	*Convallaria majalis*	*Leaf*	Bozhou, Anhui
(50)	*Disporopsis aspera*	*Herb*	Zhangjiajie, Hunan
(51)	*Disporopsis aspera*	*Leaf*	Shanghai
(52)	*Disporopsis fuscopicta*	*Root*	Bozhou, Anhui
(53)	*Disporopsis pernyi*	*Herb*	Lushan, Jiangxi
(54)	*Disporum bodinieri*	*Herb*	Lushan, Jiangxi
(55)	*Disporum calcaratum*	*Herb*	Zhangjiajie, Hunan
(56)	*Disporum sessile*	*Herb*	Lushan, Jiangxi
(57)	*Fritillaria thunbergii*	*Stem*	Shanghai
(58)	*Fritillaria cirrhosa*	*Stem*	Shanghai
(59)	*Fritillaria cirrhosa *var.* ecirrhosa*	*Stem*	Shanghai
(60)	*Fritillaria pallidiflora*	*Flower*	Bozhou, Anhui
(61)	*Hemerocallis fulva*	*Flower*	Shijiazhuang, Hebei
(62)	*Hemerocallis minor*	*Root*	Shijiazhuang, Hebei
(63)	*Hemerocallis fulva*	*Root*	Shanghai
(64)	*Heterosmilax chinensis*	*Root*	Lushan, Jiangxi
(65)	*Heterosmilax japonia*	*Leaf*	Jinyunshan, Chongqing
(66)	*Heterosmilax yunnanensis*	*Leaf*	Jingzhou, Hubei
(67)	*Hosta plantaginea*	*Flower*	Bozhou, Anhui
(68)	*Hosta plantaginea*	*Root*	Chengdu, Sichuan
(69)	*Hosta ventricosa*	*Herb*	Shanghai
(70)	*Lilium brownii*	*Herb*	Zhangjiajie, Hunan
(71)	*Lilium brownii *var.* viridulum*	*Herb*	Shanghai
(72)	*Lilium brownii *var.* viridulum*	*Flower*	Shijiazhuang, Hebei
(73)	*Lilium callosum*	*Herb*	Yunwushan, Guangdong
(74)	*Lilium longiflorum*	*Stem*	Emeishan, Sichuan
(75)	*Liriope graminifolia*	*Herb*	Shanghai
(76)	*Liriope graminifolia*	*Fruit*	Shanghai
(77)	*Liriope minor*	*Herb*	Shanghai
(78)	*Liriope platyphylla*	*Herb*	Lushan, Jiangxi
(79)	*Liriope platyphylla*	*Fruit*	Shanghai
(80)	*Liriope spicata*	*Herb*	Lushan, Jiangxi
(81)	*Notholirion hyacinthinum*	*Herb*	Chengdu, Sichuan
(82)	*Ophiopogon chingii*	*Root*	Yunwushan, Guangdong
(83)	*Ophiopogon intermedius*	*Herb*	Jingzhou, Hubei
(84)	*Ophiopogon jaburan*	*Herb*	Xishuangbanna, Yunnan
(85)	*Ophiopogon japonicus*	*Root*	Shanghai
(86)	*Ophiopogon mairei*	*Herb*	Zhangjiajie, Hunan
(87)	*Ophiopogon tonkinensis*	*Herb*	Xishuangbanna, Yunnan
(88)	*Paris chinensis*	*Herb*	Yunwushan, Guangdong
(89)	*Paris polyphylla*	*Flower*	Bozhou, Anhui
(90)	*Paris polyphylla*	*Root*	Chengdu, Sichuan
(91)	*Polygonatum cyrtonema*	*Root*	Lushan, Jiangxi
(92)	*Polygonatum cyrtonema*	*Stem*	Lushan, Jiangxi
(93)	*Polygonatum filipes*	*Leaf*	Lushan, Jiangxi
(94)	*Polygonatum filipes*	*Root*	Lushan, Jiangxi
(95)	*Polygonatum odoratum*	*Root*	Shanghai
(96)	*Polygonatum cirrhifolium*	*Root*	Chengdu, Sichuan
(97)	*Reineckia carnea*	*Herb*	Bozhou, Anhui
(98)	*Rohdea japonica*	*Root*	Bozhou, Anhui
(99)	*Rohdea japonica*	*Seeds*	Bozhou, Anhui
(100)	*Sansevieria trifasciata*	*Leaf*	Xishuangbanna, Yunnan
(101)	*Smilacina henryi*	*Herb*	Zhangjiajie, Hunan

**Table 2:** Cytotoxic effects of the extracts in the MTT assay (Hela cells and Raw 264.7 cells).

Number	Latin name	Hela cells			Raw 264.7 cells		
		Viability at 12.5 *μ*g/mL (%)	Viability at 25 *μ*g/mL (%)	Viability at 50 *μ*g/mL (%)	Viability at 12.5 *μ*g/mL (%)	Viability at 25 *μ*g/mL (%)	Viability at 50 *μ*g/mL (%)
1	*Rehderodendron macrocarpum*	105.0 ± 1.2	107.0 ± 5.2	110.5 ± 2.0	122.1 ± 2.4	129.6 ± 0.8	143.6 ± 2.5
2	*Styrax suberifolia*	87.4 ± 2.5	86.0 ± 1.9	75.8 ± 1.4	110.6 ± 1.3	99.7 ± 1.6	81.3 ± 1.9
6	*Alangium chinense *ssp.* triangulare*	103.8 ± 0.3	127.9 ± 3.3	119.4 ± 3.7	55.1 ± 2.9	55.8 ± 4.6	49.2 ± 3.9
7	*Alangium kurzii*	105.4 ± 2.0	120.8 ± 2.6	127.8 ± 4.6	130.8 ± 0.8	130.0 ± 2.2	134.2 ± 2.4
9	*Alangium kurzii *var.* handelii*	61.7 ± 1.3	49.1 ± 1.6	32.2 ± 1.2	NT	NT	NT
26	*Capparis masaikai*	110.9 ± 0.3	126.7 ± 2.0	112.3 ± 3.8	123.0 ± 1.3	126.0 ± 3.0	145.3 ± 1.8
27	*Cleome gynandra*	130.6 ± 3.3	127.9 ± 4.6	12.4 ± 0.8	132.2 ± 7.0	112.0 ± 2.1	12.1 ± 0.9
42	*Aspidistra omeiensis*	25.3 ± 1.8	09.8 ± 0.9	10.3 ± 1.0	NT	NT	NT
43	*Aspidistra omeiensis*	13.9 ± 1.9	08.9 ± 0.6	7.5 ± 0.4	NT	NT	NT
46	*Cardiocrinum cathayanum*	84.5 ± 3.5	92.8 ± 1.5	85.0 ± 2.3	121.2 ± 4.1	112.0 ± 0.8	113.2 ± 0.9
50	*Disporopsis aspera*	87.0 ± 1.7	43.5 ± 2.4	8.4 ± 0.2	NT	NT	NT
55	*Disporum calcaratum*	88.6 ± 0.8	85.9 ± 1.4	81.8 ± 4.5	80.5 ± 3.9	75.0 ± 4.8	68.0 ± 3.0
62	*Hemerocallis minor*	121.8 ± 1.8	77.9 ± 3.0	71.6 ± 4.6	118.2 ± 0.5	119.9 ± 3.2	101.6 ± 4.7
77	*Liriope minor*	128.2 ± 1.9	127.6 ± 1.4	129.1 ± 2.5	151.4 ± 0.4	179.2 ± 2.0	170.5 ± 3.9
80	*Liriope spicata*	103.4 ± 7.1	84.0 ± 3.8	64.9 ± 3.5	142.4 ± 0.5	141.9 ± 1.4	136.3 ± 1.5
85	*Ophiopogon japonicus*	81.9 ± 1.6	69.6 ± 2.6	47.7 ± 2.8	NT	NT	NT
86	*Ophiopogon mairei*	112.1 ± 2.5	93.0 ± 3.6	56.1 ± 0.9	88.4 ± 2.3	89.8 ± 0.6	76.9 ± 3.4
87	*Ophiopogon tonkinensis*	118.9 ± 3.1	79.5 ± 1.2	65.5 ± 3.6	94.0 ± 1.0	114.2 ± 4.4	104.5 ± 0.3
88	*Paris chinensis*	36.3 ± 0.3	13.0 ± 0.7	10.0 ± 1.0	NT	NT	NT
89	*Paris polyphylla*	10.6 ± 1.4	7.9 ± 0.4	5.5 ± 0.1	NT	NT	NT
90	*Paris polyphylla*	30.3 ± 0.2	6.9 ± 0.5	7.3 ± 0.8	NT	NT	NT
101	*Smilacina henryi*	63.0 ± 0.8	66.7 ± 3.4	51.3 ± 1.0	NT	NT	NT

Values are means ± standard deviations. NT: not tested.
