# Supplementary material for: An Exploration of Traditional Chinese Medicinal Plants with Anti-Inflammatory Activities
Source: Evid Based Complement Alternat Med. 2017 Apr 4;2017:1231820. doi: 10.1155/2017/1231820 (PMC5394394; doi:10.1155/2017/1231820)
Supplement: Supplementary file 1 — S1 Figure: Cytotoxicity of parthenolide in RAW 264.7 macrophages and Hela cells. Cells were incubated with parthenolid (2.5, 5, 10 and 15 μM) for 24 h. The cell viability was tested by MTT assay. The data were expressed as mean ± S.E.M. [file 1231820.f1.pdf]

SUPPORTING INFORMATION

S1 Fig

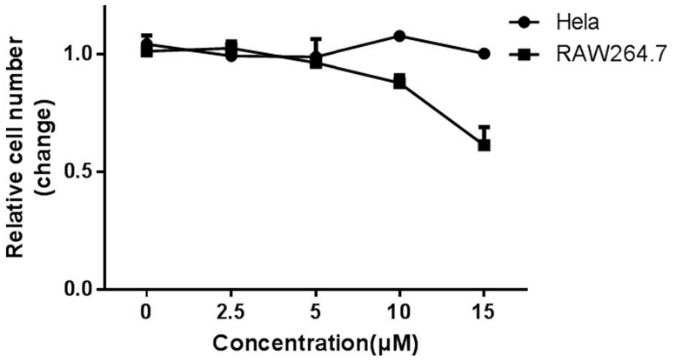

S1 Fig: Cytotoxicity of parthenolide in RAW 264.7 macrophages and Hela cells. Cells were incubated with parthenolid (2.5, 5, 10 and 15 μM) for 24 h. The cell viability was tested by MTT assay. The data were expressed as mean ±S.E.M.
